# Cell Morphology on Poly(methyl methacrylate) Microstructures as Function of Surface Energy

**DOI:** 10.1155/2019/2393481

**Published:** 2019-05-02

**Authors:** Matthias Katschnig, Boris Maroh, Natascha Andraschek, Sandra Schlögl, Ulrike Zefferer, Elisabeth Bock, Gerd Leitinger, Christa Trattnig, Maria Kaufmann, Werner Balika, Clemens Holzer, Ute Schäfer, Silke Patz

**Affiliations:** ^1^Montanuniversität Leoben, Austria; ^2^Polymer Competence Center Leoben GmbH, Austria; ^3^Research Unit for Experimental Neurotraumatology, Department of Neurosurgery, Medical University, Graz, Austria; ^4^Medical University of Graz, Gottfried Schatz Research Center, Austria; ^5^STRATEC Consumables GmbH, Austria

## Abstract

Whilst the significance of substrate topography as a regulator of cell function is well established, a systematic analysis of the principles underlying this is still unavailable. Here we evaluate the hypothesis that surface energy plays a decisive role in substrate-mediated modulation of cell phenotype by evaluation of cell behaviour on synthetic microstructures exhibiting pronounced differences in surface energy. These microstructures, specifically cubes and walls, were fabricated from a biocompatible base polymer, poly(methyl methacrylate), by variotherm injection molding. The dimensions of the cubes were 1 *μ*m x 1 *μ*m x 1 *μ*m (height x width x length) with a periodicity of 1:1 and 1:5 and the dimensions of the walls 1 *μ*m x 1 *μ*m x 15 mm (height x width x length) with a periodicity of 1:1 and 1:5. Mold inserts were made by lithography and electroplating. The surface energy of the resultant microstructures was determined by static contact angle measurements. Light scanning microscopy of the morphology of NT2/D1 and MC3T3-E1 preosteoblast cells cultured on structured PMMA samples in both cases revealed a profound surface energy dependence. “Walls” appeared to promote significant cell elongation, whilst a lack of cell adhesion was observed on “cubes” with the lowest periodicity. Contact angle measurements on walls revealed enhanced surface energy anisotropy (55 mN/m max., 10 mN/m min.) causing a lengthwise spreading of the test liquid droplet, similar to cell elongation. Surface energy measurements for cubes revealed increased isotropic hydrophobicity (87° max., H_2_O). A critical water contact angle of ≤ 80° appears to be necessary for adequate cell adhesion. A “switch” for cell adhesion and subsequently cell growth could therefore be applied by, for example, adjusting the periodicity of hydrophobic structures. In summary cell elongation on walls and a critical surface energy level for cell adhesion could be produced for NT2/D1 and MC3T3-E1 cells by symmetrical and asymmetrical energy barrier levels. We, furthermore, propose a water-drop model providing a common physicochemical cause regarding similar cell/droplet geometries and cell adhesion on the investigated microstructures.

## 1. Introduction

It is well established that cellular functions including proliferation, migration, differentiation, and motility are regulated by genetic factors, cellular communication, and chemical, metabolic, and protein-based messengers [1, 2]. Interactions with the extracellular matrix (ECM), furthermore, influence cell behaviour and have become increasingly important for the understanding of cell development [2, 3]. Alongside the impact of physicochemical matrix composition, various studies have demonstrated that topography alone can significantly modulate cell function [4–7].

The three-dimensional topography of the ECM is created by interwoven fibrillar proteins (e.g., collagens, elastins, fibronectins, and laminins) embedded within a network of proteoglycans [2, 8]. The proteins of the ECM and basement membrane, as well as their interconnecting pores, exhibit microscale and nanoscale dimensions [9] and collectively produce a complex environment with hierarchically structured microscale and nanoscale pores, pillars, grooves, and ridges [10, 11]. The ECM, moreover, contains nonmatrix proteins, such as soluble growth factors. Together these features of the ECM provide mechanical, chemical, and physical cues that modulate cell behaviour and functionality [9, 12, 13].

Interventional modulation of specific cell functions through the use of synthetic micro- and nanostructures with architecture and geometry mimicking specific features of the ECM is an attractive idea. Microstructures could thereby be categorized by a dimensional scale above 1 *μ*m and nanostructures beyond 1 *μ*m. Several studies have shown that interaction of a variety of cell types with micro- and nanostructures generally results in increased adhesion and proliferation. This has been shown for fibroblasts, smooth muscle cells, endothelial cells, osteoblasts, and mesenchymal stem cells cultured on islands [14–16], columns [17], fibres [18–22], or angular structures [23].

We hypothesized that variable surface energy associated with different surface topographies plays a decisive role in the observed differential modulation of cell behaviour. To test this, we created polymeric microstructures with pronounced differences in surface energy. These are basically manufactured on an industrial scale, mainly by hot embossing [24–28] or injection molding [27, 29–34]. The former is defined as the stamping of a pattern into a softened polymer at elevated temperatures. Its main advantages are precise replication of even nanotopographies and low material strain due to low shearing. Its main disadvantage is a long cycle time up to half an hour.

Injection molding comprises the injection of molten plastic into a mold under high pressure. The material for the part is fed into a heated barrel, molten and mixed, and forced into a mold cavity, where it is cooled and solidified to the exact shape of the cavity [35]. For the replication of microstructured surfaces, the standard injection molding process is adapted using a microstructured stamper as a mold cavity insert fixed by a frame. The main advantages of injection molding are high replication numbers at low cycle times and precise microstructure demolding, whilst the main disadvantages are high machine and mold costs.

To achieve large numbers of products, both techniques produce replicates of the so-called stamper (the negative of the desired structure) many times. The stampers can be fabricated in several ways, including micromachining by CNC (computerized numerical control) milling and different forms of LIGA (lithography electroplating and molding).

Due to the fast freezing of the polymer melt filling the negative topography and thus a lack of demolding quality, a variotherm system (rapid heating/cooling system of the mold) is commonly used to create microstructures by injection molding [27, 31, 36–38]. The basic variotherm process usually comprises rapid elevation of mold temperature above the glass transition temperature of polymers to extend the liquid period of polymer melts during the filling phase [39]. Different types of variotherm process have been developed, for instance, incorporating inductive heating or electrical resistance heating with cooling by liquid cold media [39].

## 2. Materials and Methods

### 2.1. Base Polymer Selection

Poly(methyl methacrylate) (PMMA), also known by the trade name Plexiglas©, was selected as polymeric cell niche based on our previous work [40]. PMMA generally exhibits hydrophilic characteristics due to the ester groups in its macromolecular backbone (molecular formula: C_5_O_2_H_8_)_n_), is sufficiently and optically transparent to permit cell observation by microscopy, and exhibits good casting qualities for microstructuring [41]. Medical grade material (DELPET 70NH, Asahi Kasei Chemicals Corporation, Japan) delivered in granule form and ready for injection molding was used. The melt flow rate is reported to be 1.8 g/10 min (230°C, 3.8 kg) and the bulk density 1.19 g/cm^3^. The tensile modulus is reported to be 3300 MPa and the tensile strength at break 67 MPa; thus the material is very stiff. The recommended barrel (210°C-250°C) and mold temperatures (50°C-70°C) were observed. Prior to molding, the material was predried at 90°C for 4 h using a dehumidifying drier. Standard polystyrene (PS) cell culture dishes were used for reference purposes.

### 2.2. Mold Insert Fabrication

Nickel mold inserts comprising the microstructure negative were fabricated by the LIGA process and remained uncoated and untreated to avoid sample contamination.

### 2.3. Variotherm Injection Molding

Microstructure fabrication was performed at STRATEC Consumables GmbH (Former Sony DADC BioSciences GmbH, Austria) in a clean room environment by injection compression molding using a fully electric injection molding machine (ENGEL e-motion 100, ENGEL AUSTRIA GmbH, Austria). The mold used featured a hot runner system and was evacuated before injection. A company-internal standard process parameter set optimized for optical clarity and replication of structures was deployed.

A variotherm process was implemented by electrical resistance heating based on previous results showing that variotherm processes are very useful for high aspect ratio microstructure replication [39, 42, 43]. Molded slide sterility was maintained by aseptic packaging in sterile containers.

### 2.4. Evaluation of Demolding Quality and Slide Holding Concept

Demolding quality of the injection molded slides was evaluated by Scanning Electron Microscopy (SEM) and Atomic Force Microscopy (AFM). For SEM we used a Zeiss DSM950 scanning electron microscope (Carl Zeiss AG, Germany) employing a secondary electron detector and an acceleration voltage of 10 kV. Polymer samples were sputter-coated with gold to ensure surface conductivity. The employed sputter coater was a Bal-Tec SCD 500 (Capovani Brothers Inc., USA) and the sputtered gold layer thickness was approximately 15 nm based on sputter time (60 s) and current (40 mA). Postdata processing was performed using Paint.NET© v4 (dotPDN LLC, USA). For AFM we used a Dimension 3100 Series AFM (Digital Instruments Incorporated, USA) in tapping mode. The respective scan area was 5 x 5 *μ*m^2^; evaluated area was 4 x 4 *μ*m^2^ to avoid fringe effects. Tip velocity was 10 *μ*m/s, average amplitude set point 1.5 V, and average drive amplitude 250 mV. As cantilever tip, a Veeco OTESPA Wafer silicon probe (k=42 N/m, f_0_=300 kHz) (Asylum Research, USA) for general samples, was used. Data was collected using NanoScope Version 5.12 (Digital Instruments Veeco Metrology Group, USA) and processed using Gwyddion v2.26 (Czech Metrology Institute, Czech Republic). The slides were finally incorporated into a Millipore® Millicell™ slide adapter (Merck KgaA, Germany) for cell culture applications (Figure S1B-C).

### 2.5. Contact Angle and Surface Energy Measurements

Water contact angles are commonly used to measure overall surface wettability, whilst surface energy differentiates between wettability caused by polar groups and that by disperse forces such as van der Waals attractions.

Contact angle measurements were carried out with a DSA 100 Drop Shape Analysis System (Krüss, Germany) using deionized water and diiodomethane as test liquids. Droplet volume for both test liquids was 2 *μ*L. In each experiment, a drop was applied to a solid sample (sessile drop) and a cross-sectional image of the drop was captured with a camera and transferred to the drop shape analysis software. Contour recognition was initially carried out based on a grey-scale analysis of the image. In the second step, a geometrical model describing the drop shape was applied to fit the contour of the drop. The Young-Laplace-Fit, which is applicable to ideal sessile drops flattened by their own weight and for contact angles in the range of 10° to 180°, was thus chosen as basic method. For highly symmetric drop geometry, the polynomial method was preferred [44]. Mean contact angle values were calculated from at least ten individual measurements.

Surface energy was then calculated according to Owens, Wendt, Rabel, and Kaelble [45, 46] using the software DSA1 v1.9 (Krüss, Germany). To model and determine the wetting state on the surface, that is, Wenzel regime or Cassie-Baxter regime, we also studied the wetting behaviour of smaller (1 *μ*L) and larger (5 *μ*L) deionized water droplets. Mean contact angle values were again calculated from at least ten individual measurements. Reproducibility was assured by a maximum standard deviation of ±3°. A nonstructured PMMA slide served as reference. Surface energy calculation software did not facilitate error propagation, so no standard deviation could be displayed.

### 2.6. UV Sterilization

Ultraviolet (UV) light sterilization for cell culture investigations (1.7 mW/cm^2^ for 1 hour) was carried out using a Microbiological Safety Class II Workbench KS9 (Heraeus Holding GmbH, Germany).

### 2.7. Cell Culture

5 x 10^3^ NT2/D1 or MC3T3-E1 cells were seeded into 1 cm^2^ wells on either structured or nonstructured PMMA provided by STRATEC Consumables GmbH. NT2/D1 cells were cultured in Dulbecco's modified Eagle's medium supplemented with 10% fetal bovine serum (FBS) at 37°C in 95% air/5% CO_2_. MC3T3-E1 cells were cultured in MEM containing 10% FBS and 2 mM Glutamate at 37°C in 95% air/5% CO_2_. Both cell lines were grown to 70–90% confluence.

### 2.8. Cell Staining and Microscopy

For F-actin staining, NT2/D1 and MC3T3-E1 cells were washed twice with PBS following culture for 48 hrs and subsequently fixed with 3% paraformaldehyde for 10 min at room temperature (RT) and permeabilized with 0.1% Triton-X100 for 5 min at RT. Rhodamine-phalloidin stock solution in methanol with a concentration of 6.6 *μ*M (300 units/mL) (540/565 nm; Invitrogen) was diluted in PBS (5 *μ*L stock in 200 *μ*L PBS) and incubated for 20 min at RT to stain F-actin bundles. Nuclei were visualized by DAPI counterstaining for 5 min at RT (stock solution concentration of 5 mg/mL, 10.9 mM, and working solution concentration of 300 nM) (358/461 nm, Invitrogen). Laser confocal imaging (Zeiss SM 510) was performed using a 10x objective and an excitation wavelength of 488 nm and 543 nm with an open aperture.

### 2.9. Gene Expression Analyses

Cells were cultured as described above, snap-frozen in liquid nitrogen, and stored at -80°C for further analysis. Total RNA was isolated using the RNeasy micro Kit (Qiagen, Germany), according to the manufacturer's instructions. Following RNA quality control by 1.2% formaldehyde agarose gel electrophoresis, cDNA was synthesized from 200 ng total RNA using the Fermentas First Aid cDNA First Strand Synthesis Kit (Thermo Fisher Scientific, Germany) according to the manufacturer's instructions.* Amplicon sizes and primer sequences*: NF200 (160 bp), forward: 3'-GAGGAACACCAAGTGGGAGA-5'; reverse: 3'-TTCTGGAAGCGAGAAAGGAA -5'; MAP (319 bp), forward: 3'-TCAGAGGCAATGACCTTACC-5'; reverse: 3'-GTGGTAGGCTCTTGGTCTTT-5'; Tuj1 (359 bp), forward: 3'- GGCAACCAGATCGGGGCCAAGT-5'; reverse: 3'-CCCTGCAGGCAGTCGCAGTTT-5'; U6 (94 bp), forward: 3'-CTCGCTTCGGCAGCACA-5'; reverse: 3'-AACGCTTCACGAATTTGCGT-5'.* PCR conditions*: denaturation at 95°C for 5 min followed by 35 cycles of amplification for NF200 (30 s, 60°C), 32 cycles for MAP2 (30 s, 58°C), 35 cycles for Tuj1 (30 s, 60°C), and 35 cycles for U6 (30 s, 60°C). Denaturation (95°C), annealing, and extension (72°C) times were all 30 s. Final extension was at 72°C for 10 min, PCR products were resolved by 2% agarose gel electrophoresis, and densitometric analysis was performed with Quantity One software (Bio-Rad, Hercules, CA, USA). U6 was used as reference gene.

## 3. Results and Discussion

### 3.1. Structure Design

We hypothesized that local variations of surface energy by different microstructures play a decisive role in the modulation of the investigated cell types. This hypothesis is based on prior publications; for instance, a surface energy influence on fibroblast growth and spreading was shown by Schakenraad et al. [47] and on osteoblast adhesion by Ranella et al. [48]. Our first step in testing this hypothesis was, thus, to establish microstructures with pronounced differences in surface energy.

**Figure 1:**
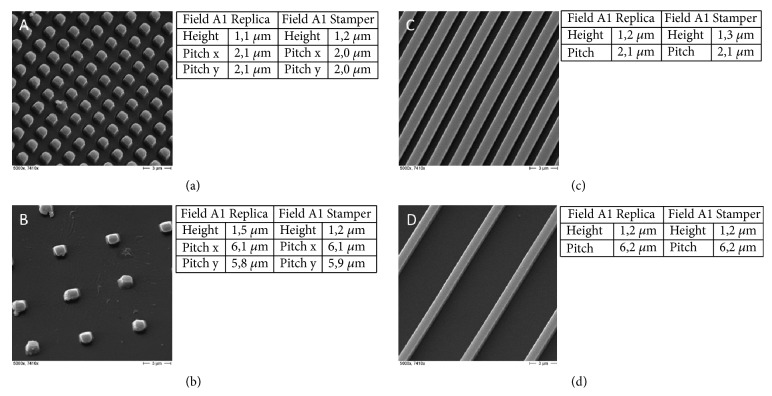
(a)-(d): SEM images of the injection-molded microstructures (left images) and the AFM profile measurements of the injection-molded microstructures compared according to the stamper (right tables). Pitch measured from center point to center point of structure elements. (a) Cubes P1:1; (b) cubes P1:5; (c) walls P1:1; (d) walls P1:5.

Cubes and walls were selected to underlie microstructure design based on already published knowledge and technical considerations regarding proper demolding. The dimensions of the cubes were 1 *μ*m x 1 *μ*m x 1 *μ*m (height x width x length) with a periodicity of 1:1 and 1:5 and the dimensions of the walls 1 *μ*m x 1 *μ*m x 15 mm (height x width x length) with a periodicity of 1:1 and 1:5. The microstructures were located on a standard microscopy slide of 1 mm x 25 mm x 75 mm (height x width x length) on an area of 150 mm^2^ for each structure field (Figure S1A). The injection-molded slides were examined by SEM and AFM to confirm adequate demolding quality. In general, PMMA is less suitable for proper demolding because of the material's polar nature, which causes it to stick to the stamper. Adequate demolding quality was, nevertheless, achieved even without mold release agents or mold coating, as shown by a comparison of stamper and replica (Figures 1(a)–1(d)).

### 3.2. Evaluation of Surface Energy/Drop Shape Anisotropy

Parallel (“pa”) and perpendicular (“pe”) static contact angles to the microstructure were measured to verify the assumed asymmetry of surface energy on microstructures. The microstructure “walls” was, thereby, used as coordinate reference: along walls it was defined as “parallel” (0°) and across walls it was defined as “perpendicular” (90°).

**Figure 2:**
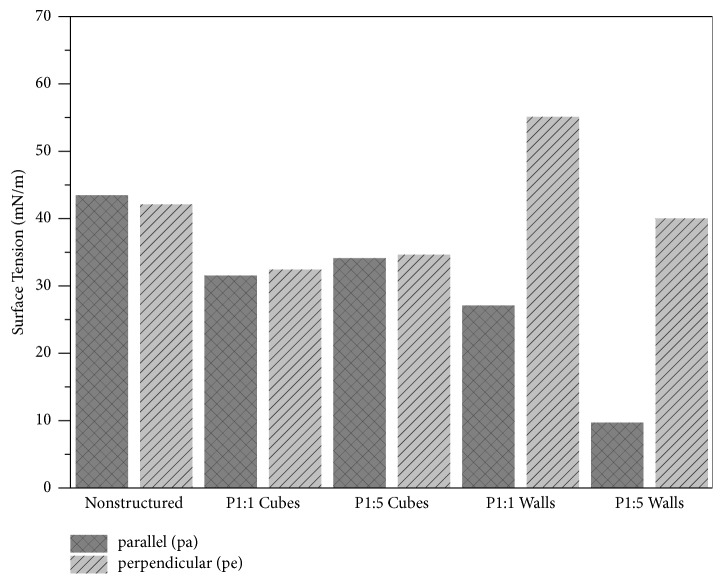
Surface tension depends on substrate topography. Calculated surface energy as function of substrate topography. Contact angles were measured perpendicular or parallel to nonstructured and specific surface structures.

Drop shape anisotropy with respect to surface energy means that contact angles differ depending on the measurement direction. The received contact angles and the derived surface energy values of water and diiodomethane are listed in Table 1; surface energy values alone are shown in Figure 2.

Surfaces with cubes (P1:1, P1:5) and the nonstructured reference surface exhibited “symmetric droplets” indicative of isotropic wetting properties (discontinuous design). Nonstructured reference exhibited typical contact angles as given in [49]. The total surface energy of the PMMA substrate was, however, significantly decreased by the introduction of cubic structures. For P1:5 structures, this decrease was mainly related to a depletion of the dispersing surface energy component. With lower cube spacing (P1:1), the decrease in total surface energy was even more pronounced, since both polar and disperse components were decreased.

In contrast to control and cubes, for which we observed similar wetting in both parallel and perpendicular directions, the wetting properties of wall structures were anisotropic (continuous design), as the contact angles of both test liquids exhibited lower surface energy (hydrophobic properties) parallel to wall direction and higher values (hydrophilic properties) perpendicular to wall structures (Figure 2, Table 1). Particularly for P1:1 walls the water contact angle was 132° perpendicularly to the structures, whilst it was 59° parallel to the structure.

**Figure 3:**
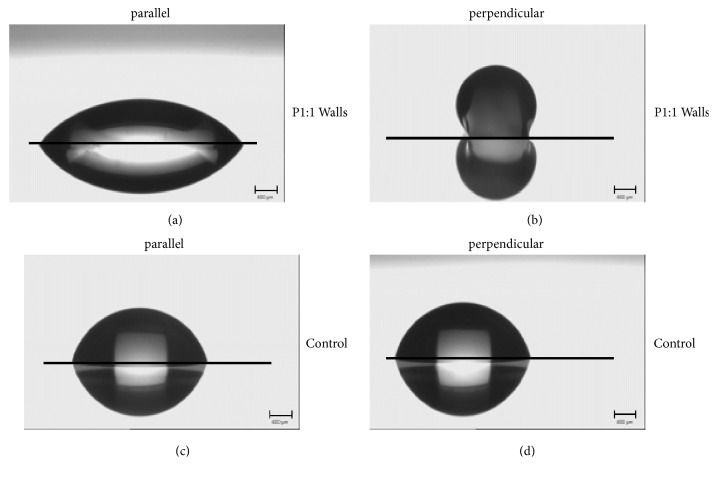
Energy optimized model of water droplets. Behaviour of water droplets on nonstructured control and P1:1 walls. (a) Water droplet parallel to P1:1 walls; (b) water droplet perpendicular to P1:1 walls; (c) water droplet on nonstructured control, parallel; (d) water droplet on nonstructured control, perpendicular.

The results shown in Table 1 suggest that test liquid droplet spreading along the lengthwise wall direction, which exhibits the higher surface energy, was energetically favoured. In other words, the test liquid tends to wet the structure in a manner similar to a “capillary effect” parallel to walls (Figure 3(a)). The perpendicular direction, in contrast, represents an energy barrier with the low surface energy of the structure as derived from Owens, Wendt, Rabel, and Kaelble calculations. The test liquid consequently forms an energy-optimized spherical geometry based on a “repelling effect” and the droplet is pinned (Figure 3(b)). These anisotropic energy barriers were not observed on the nonstructured control, on which the droplet formed similar shapes for both parallel and perpendicular measurements, indicating isotropic surface energy of the substrate (Figures 3(c)-3(d)).

**Figure 4:**
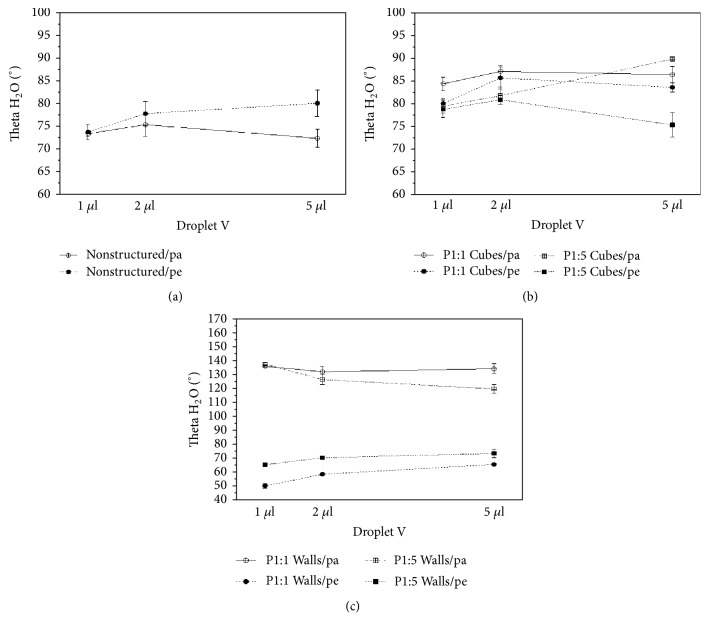
Water contact angles as function of droplet volume and substrate topography. (a) Nonstructured control, (b) cubes, and (c) walls.

Based on the theoretical considerations presented in [50, 51], different droplet volumes (1 to 5 *μ*L) were used to define the wetting state of a water droplet on microstructured samples. The contact angles obtained are listed in Table 2 and displayed in Figure 4 as a function of droplet volume and substrate topography. With respect to the nonstructured PMMA surface, anisotropy was significant with 5 *μ*L of the test liquid (H_2_O, pe: 72°, pa: 80°) in comparison to 1 *μ*L of the test liquid (H_2_O, pe: 73°, pa: 74°). No significant anisotropy was observed on the surface with P1:1 cubic structure with increasing droplet volumes. However, with rising pitch (P1:5), the structured surface showed the same insignificant trend in anisotropy (H_2_O, pe: 90°, pa: 76°) as the nonstructured control. Walls exhibited significant anisotropy with the investigated drop volumes. Here we observed that structures with a smaller pitch exhibited higher anisotropy than structures with a larger pitch, whilst anisotropy was more pronounced with a smaller test liquid volume.

The results suggest that wetting behaviour on structured samples follows either a Wenzel (complete topography filling by test liquid) or a mixed wetting regimen (incomplete filling of topography by test liquid). A further indication is provided by drops “sticking” to the structured surface when the substrate samples are tilted (at 45° and 90° angles) as suggested by Neuhaus et al. [51]. In the case of a full Cassie-Baxter regime, a drop with air trapped beneath it would slide off the surface due to a composite air-polymer substrate and thus reduced physical attachment to the (polar) polymer surface [52].

### 3.3. Cell Shape is Modulated by Microstructures

Specific drop shapes linked to a substrate by intermolecular forces are well documented for wetting regimens of liquids on heterogeneous surfaces [50–52]. According to Alberts et al. [2], changes in and of optimization of cell shape are driven by a minimization of total free energy and of the surface-to-volume ratio of the cell membrane. We therefore hypothesize that cells correlate their focal adhesion network and subsequently their shape on heterogeneous surfaces in a manner analogous to water drops with differences in boundary surface energy “energy barriers” to minimize total free energy. These energy barriers are also denoted by the above described contact angle measurements on nonstructured controls and structured PMMA substrates (Table 1).

**Figure 5:**
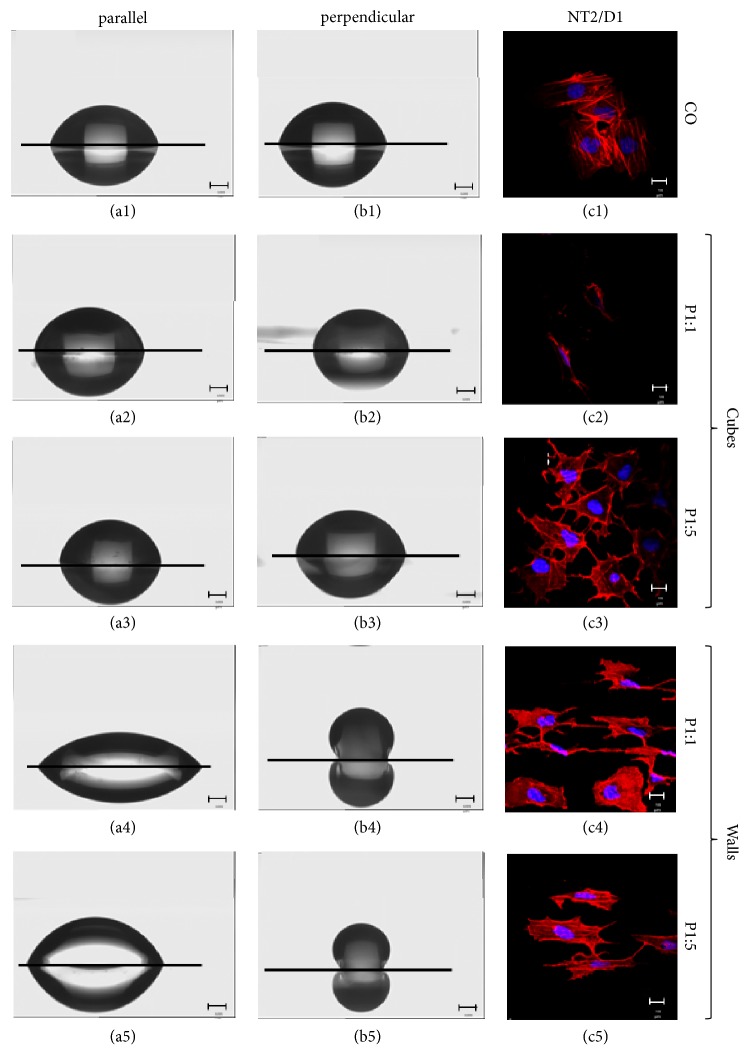
The water drop model. Comparison between water droplet shapes and cell morphology of NT2/D1. (a1)-(a5): side view, parallel to walls reference; (b1)-(b5): side view, perpendicular to walls reference; (c1)-(c5): NT2/D1 cells in LSM top view on microstructures. Microstructures indicated on the right side. Scale bar=10 *µ*m.

The pluripotent human embryonal carcinoma cell line NTera2/cl.d1 (NT2/D1) which is able to differentiate into mature neurons [53] was chosen as model to test this. NT2/D1 precursors are commonly used as a model for neurogenesis [40] and have already shown promise for implementation in experimental [54–56] and clinical [57] cell replacement approaches. For a clearer presentation of our results we show a clear contrast between parallel (Figures 5(a1)–5(a5)) and perpendicular (Figures 5(b1)–5(b5)) water droplet behaviour and our observed NT2/D1 morphological changes (Figures 5(c1)–5(c5)) on nonstructured control and microstructured PMMA substrates.

We observed striking PMMA structure-dependent morphological differences. Cells cultured on cubes P1:1, unlike those on the unstructured substrate, exhibited poor adherence. The few cells attached to the surface appeared “out of shape” indicating a loss of cellular integrity pointing to a repelling action of this surface structure (Figure 5(c2)). On cubic structure P1:5, cells appeared triangular or quadrangular shaped, sitting on the given cubes (Figure 5(c3)). Cells cultured on walls P1:1 and P1:5 appeared quadrangular and elongated, with alignment of the cytoskeleton along the underlying walls (Figures 5(c4)+5(c5)).

At first sight, the quadrangular, elongated form adopted by NT2/D1 cells on wall-structured substrates appeared to resemble the morphological features of a more mature neuronal cell. We therefore evaluated the expression by these cells of three markers of advanced neuronal structural differentiation, namely, MAP2, NF200, and Tuj1 (ßIII Tubulin). None of these markers was, however, elevated in response to the different microstructured surfaces (Figure S2), eliminating an occurrence of molecular neuronal differentiation, during the observed time window, in contrast to the results reported by other groups [58–63]. We could not determine an involvement of different substrate microtopographies on cell differentiation as suggested, for example, by Schernthaner et al., for nanotopographies [64, 65]. However, these results were generated using endothelial cells; therefore we cannot directly compare different cells types and possible cell type related responses. Moreover, neuronal differentiation on structured surfaces would, however, probably need more time, since chemical induction of neuronal differentiation of NT2/D1 cells with retinoic acid takes around 6 weeks to produce fully differentiated neurons. Cultivation of undifferentiated cells for 48 h on the investigated structures thus appears unlikely to induce real effects on differentiation. The observed morphological changes were nevertheless profound and consequently do at least support the notion that cells per se respond to the underlying topography with morphological changes as previously described [3, 63, 66–72].

**Figure 6:**
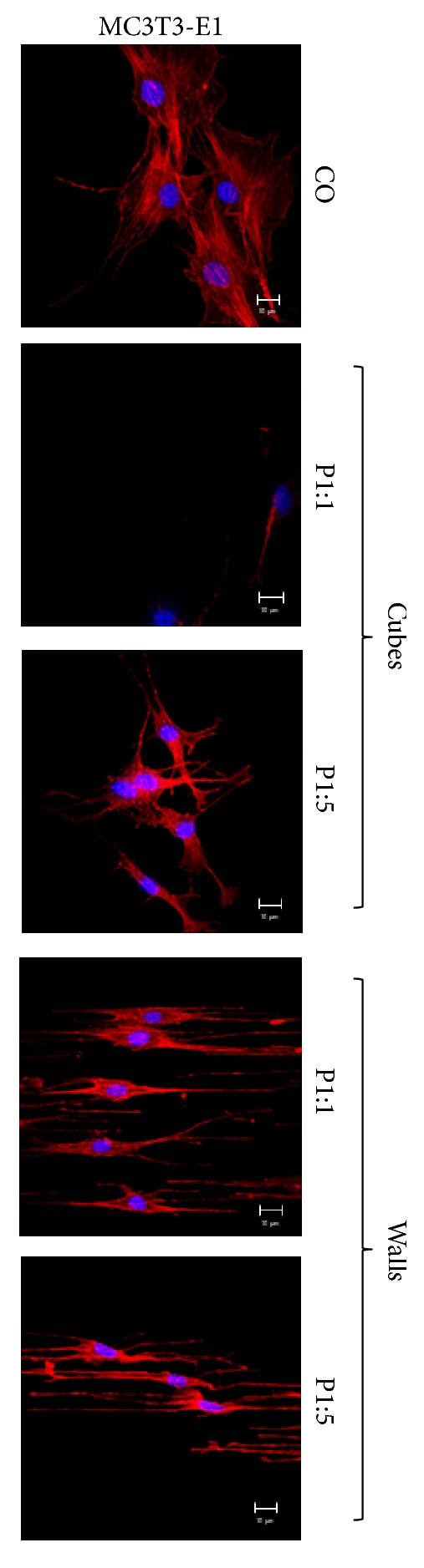
Cell morphology of MC3T3-E1 cells changes in response to the structured surface. LSM images of MC3T3-E1 cells on microstructures indicated on the right side. Scale bar=10 *µ*m.

We therefore hypothesized that other cell types would respond similarly to substrate structure as also postulated by Janson et al. [67]. To test this, we cultivated a mouse preosteoblast cell line MC3T3-E1 that is used to model ECM induced osteoblast modulation on structured and nonstructured PMMA substrates. As shown in Figure 6 the morphological changes described above for NT2/D1 cells on the structured substrates were also observed with MC3T3-E1 cells. Although osteoblasts are considered robust, these also exhibited poorer adherence to the cubic structure P1:1 than to the unstructured surface, with only a few inchoate-shaped cells sticking to the surface, underlining the previously observed repellent action of this surface structure. Cells cultured on cube P1:5, in contrast, appeared triangular to quadrangular and more bipolar-shaped/elongated on walls P1:1 and P1:5. The intracellular network of interlinking filaments and tubules of the cytoskeleton again were adjusted to the given wall structure. Whilst the results were similar for both cell lines and thus support a cell type-independent mechanism, the observed elongation was noticeably more pronounced with MC3T3-E1 preosteoblasts than with NT2/D1 cells.

We thus interpret this to indicate that the mechanism underlying the pronounced elongation of MC3T3-E1 and NT2/D1 cells on walls is fundamentally the same as that causing the spreading of water droplets, that is, asymmetric energy barrier heights, displayed, for example, in a difference, between surface energies perpendicular (55.1 mN/m) and parallel (27.1 mN/m) to walls P1:1 (Table 1).

An average isotropic water contact angle of ≤ 85° and an average isotropic surface energy of ≥ 32 mN/m appear to be necessary for effective MC3T3-E1 and NT2/D1 cell adhesion, accounting for the repellent action of cubes P1:1 observed with both cell lines tested. Based on this, we postulate a “switch” for cell adhesion and subsequently cell growth through adjustment of cube periodicity (P1:1 → P1:5) and consequently increased hydrophobicity.

Our simplified “water-drop model” presented here could describe a common physicochemical cause regarding the similar cell and droplet geometry observed on microstructures and nonstructured control and the cell adhesion of MC3T3-E1 and NT2/D1 cells. It seems that both phenomena appear to be based on surface energy barriers that differ in both direction and strength, as also described by Janson et al. [67]. The model could possibly account for the cell phenomena guided by anisotropic topographical cues discussed by Davidenko et al. [73], Mitchell et al. [74], and Thomson et al. [75] among other studies.

## 4. Conclusion

The aim of the present study was to gain basic knowledge of component behaviour on polymeric microstructures mimicking extracellular matrix topography. We thereby hypothesized a fundamental role in this for variable substrate surface energy associated with different surface topographies and consequently designed and investigated microstructures with pronounced differences in surface energy. PMMA was chosen as base polymer, structure design included cubes and walls at the micrometre scale, and structuring was established in appropriate demolding quality by injection molding.

Our results indicated no influence of microstructures on MC3T3-E1 and NT2/D1 cell differentiation within the observed time frame. A profound impact on adhesion and morphology was, however, observed, which was related to the influence of boundary surface energy. Comparison of two different cell types produced similar results, supporting cell-independent causality. For adequate adhesion, a maximisation of average isotropic water contact angle as well as minimisation of average isotropic surface energy appeared to be necessary. Thus, a “switch” to cell adhesion and subsequently to cell growth could be applied by adjusting cube periodicity. Contact angle measurements on the microstructures demonstrated enhanced surface energy anisotropy on wall-structured surfaces causing lengthwise spreading of the test liquid droplet analogous to cell elongation. Both phenomena appear to be based on variable surface energy barriers in direction and strength.

We, hence, propose a water drop model potentially providing a common physicochemical cause regarding MC3T3-E1 and NT2/D1 cell adhesion and similar cell and droplet geometry on microstructures and nonstructured control.

## Figures and Tables

**Table 1:** Contact angles and calculated surface energy values for structured and nonstructured (control) surfaces (pe: perpendicular, pa: parallel).

	H_2_O (°)	CH_2_I_2_ (°)	Surface energy (mN/m)	Polar part (mN/m)	Disperse part (mN/m)
Nonstructured/pa	75.4±2.6	43.5±2.9	43.4	5.6	37.8

Nonstructured/pe	77.8±2.7	44.6±3.0	42.0	4.8	37.3

P1:1 Cubes/pa	87.2±1.3	61.4±2.8	31.4	3.6	27.8

P1:1 Cubes/pe	85.8±2.4	60.3±2.5	32.4	3.9	28.4

P1:5 Cubes/pa	81.9±1.9	59.8±1.9	34.0	5.3	28.7

P1:5 Cubes/pe	81.0±0.9	59.3±2.7	34.6	5.6	29.0

P1:1 Walls/pa	131.9±3.9	66.8±0.3	27.1	2.4	24.7

P1:1 Walls/pe	58.6±0.7	32.9±1.4	55.0	12.0	43.0

P1:5 Walls/pa	126.4±3,7	97.4±2.8	9.6	0.0	9.6

P1:5 Walls/pe	70.4±1.4	58.5±1.4	40.1	10.6	29.4

**Table 2:** Contact angles of water as a function of droplet volume and substrate topography.

	1 *µ*L H_2_O (°)	2 *µ*L H_2_O (°)	5 *µ*L H_2_O (°)
Nonstructured/pa	73.4±0.6	75.4±2.6	72.4±2.0

Nonstructured/pe	73.8±1.6	77.8±2.7	80.1±2.9

P1:1 Cubes/pa	84.5±1.4	87.2±1.3	86.5±1.8

P1:1 Cubes/pe	80.2±1.2	85.8±2.4	83.7±1.0

P1:5 Cubes/pa	79.6±1.5	81.9±1.9	89.9±0.5

P1:5 Cubes/pe	78.9±1.9	81.0±0.9	75.5±2.7

P1:1 Walls/pa	135.9±0.8	131.9±3.9	134.1±3.5

P1:1 Walls/pe	50.3±1.8	58.6±0.7	65.6±0.8

P1:5 Walls/pa	137.4±1.0	126.4±3.7	119.6±3.0

P1:5 Walls/pe	65.5±1.5	70.4±1.1	73.5±3.1

## Data Availability

The data used to support the findings of this study are available from the corresponding author upon request.

## Supplementary Materials

Supplementary Materials Supplementary Figure 1: test system for cell laboratory. A: 4-panel-slide with structure fields (left) and without structure fields as nanostructured control (right). B: 4-panelslide with Millipore adapter. C: assembled test system. Supplementary Figure 2: analysis of differentiation marker by RT-PCR. mRNA expression of A: NF200, B: MAP2, and C: Tuj1 in response to nonstructured control and structures as indicated in the figure. n=3 biological replicates; error bars show standard error of the mean (SEM).
