# Supplementary material for: Cell Morphology on Poly(methyl methacrylate) Microstructures as Function of Surface Energy
Source: Int J Biomater. 2019 May 2;2019:2393481. doi: 10.1155/2019/2393481 (PMC6521382; doi:10.1155/2019/2393481)
Supplement: Supplementary Materials — Supplementary Figure 1: test system for cell laboratory. A: 4-panel-slide with structure fields (left) and without structure fields as nanostructured control (right). B: 4-panelslide with Millipore adapter. C: assembled test system. Supplementary Figure 2: analysis of differentiation marker by RT-PCR. mRNA expression of A: NF200, B: MAP2, and C: Tuj1 in response to nonstructured control and structures as indicated in the figure. n=3 biological replicates; error bars show standard error of the mean (SEM). [file 2393481.f1.docx]

**Supplementary Material**

**
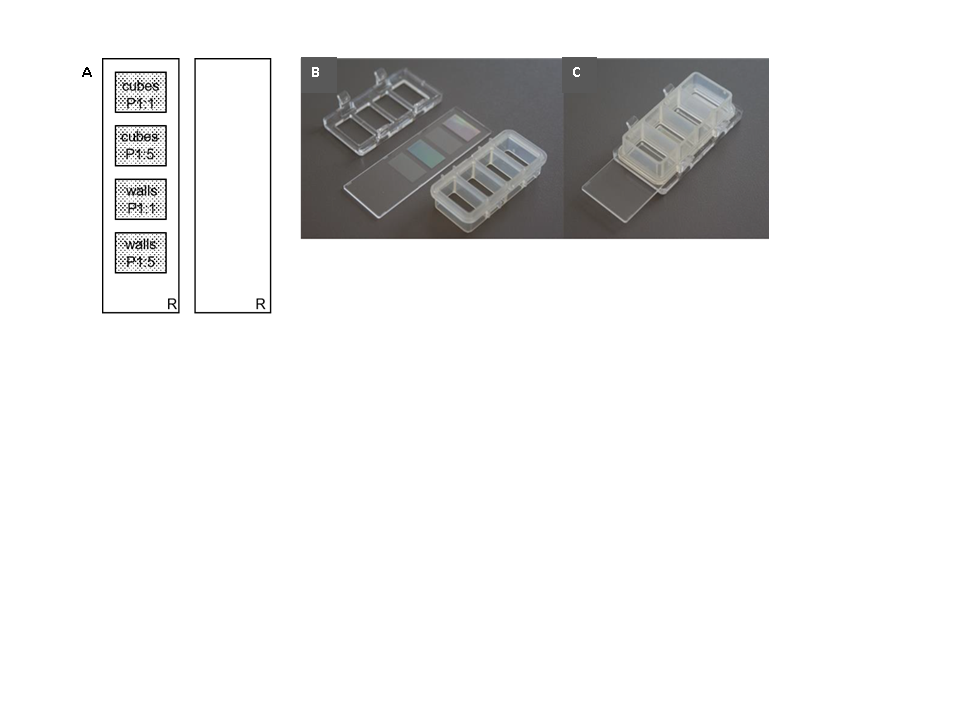
**

**Supplementary Fig. 1:** Test system for cell laboratory. **A**: 4-panel-slide with structure fields (left) and without structure fields as nanostructured control (right). **B**: 4-panel-slide with Millipore adapter. **C**: assembled test system.


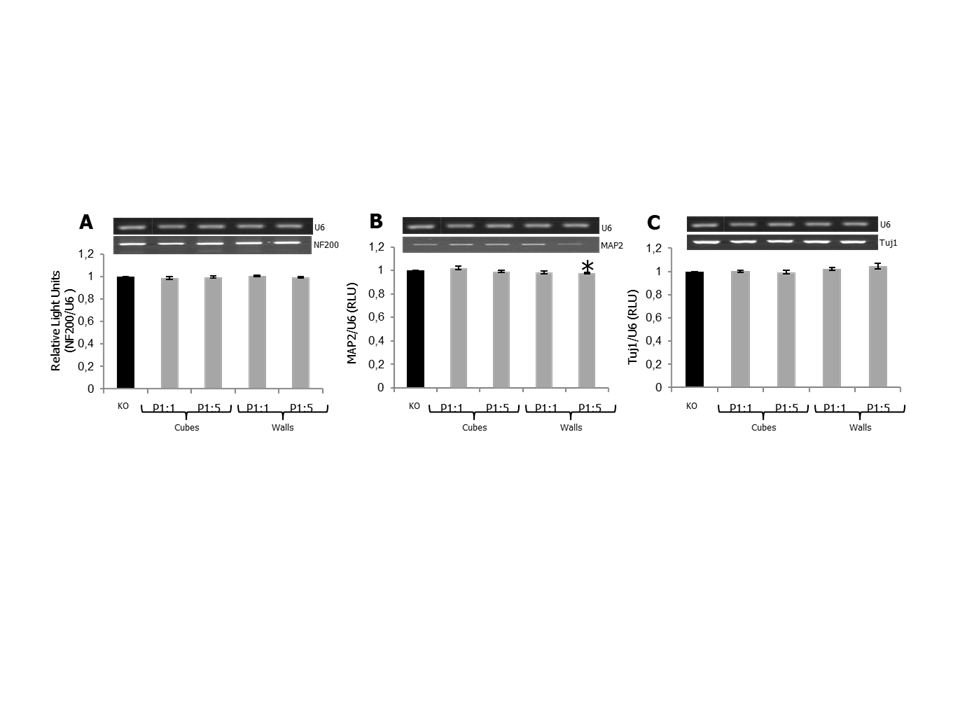
**Supplementary Fig. 2**: Analysis of differentiation marker by RT-PCR.

mRNA expression of **A**: NF200, **B**: MAP2 and **C**: Tuj1 in response to non-structured control and structures as indicated in the Figure. *n=3* biological replicates; Error bars show standard error of the mean (SEM).
